# Older Adults Living in Disadvantaged Areas: Protocol for a Mixed Methods Baseline Study on Homes, Quality of Life, and Participation in Transitioning Neighborhoods

**DOI:** 10.2196/41255

**Published:** 2022-10-12

**Authors:** Marianne Granbom, Håkan Jönson, Anders Kottorp

**Affiliations:** 1 Department of Health Sciences Lund University Lund Sweden; 2 School of Social Work Lund University Lund Sweden; 3 Faculty of Health and Society Malmö University Malmö Sweden

**Keywords:** aging, housing, neighborhood, quality of life, participation, rural areas, disadvantaged areas, aging-in-place, relocation, socioeconomic status, older adults, elderly, mental health, physical health, social participation, health dynamics

## Abstract

**Background:**

Swedish policy states that older adults should be able to age safely with continued independence and lead active lives. However, this plays out differently in different Swedish municipalities depending upon degree of demographic change, globalization, and urbanization. Internationally, older adults living in disadvantaged areas have worse physical and mental health, activity restrictions, and reduced life expectancy. In Sweden, research on how disadvantaged areas impact older adults’ quality of life is virtually nonexistent. We argue that disadvantaged areas exist in both urban and rural contexts.

**Objective:**

We aimed to investigate how older adults’ homes and neighborhoods influence their community participation, quality of life, identity, and belonging in urban and rural disadvantaged areas in Sweden, and how these person–context dynamics are experienced by older adults in transitioning neighborhoods.

**Methods:**

The study has a mixed methods design and includes 3 phases. Adults 65 years and older living in certain urban and rural disadvantaged areas in the south of Sweden will be included. Phase 1 is an interview study in which qualitative data are collected on neighborhood attachment, identity, and belonging through semistructured interviews and photo-elicitation interviews with 40 subjects. A variety of qualitative data analysis procedures are used. In phase 2, a survey study will be conducted to explore associations between observable and self-rated aspects of housing and neighborhood (physical, social, and emotional), participation, and quality of life; 400 subjects will be recruited and added to the 40 phase-1 subjects for a total of 440. The survey will include standardized measures and study-specific questions. Survey data will be analyzed with mainstream statistical analyses and structural equation modeling to understand the interactions between quality of life, home and neighborhood factors, and sociodemographic factors. In phase 3, the integration study, survey data from the 40 participants who participated in both data collections will be analyzed together with qualitative data with a mixed methods analysis approach.

**Results:**

As of the submission of this protocol (August 2022), recruitment for the interview study is complete (N=39), and 267 participants have been recruited and have completed data collection in the survey study. We expect recruitment and data collection to be finalized by December 2022.

**Conclusions:**

With an increasing proportion of older adults, an increasing number of disadvantaged areas, and an increasing dependency ratio in more than 50% of Swedish municipalities, these municipalities are transforming and becoming increasingly segregated. This study will add unique knowledge on what it is like to be older in a disadvantaged area and deepen knowledge on housing and health dynamics in later life. Further, the design of the current study will allow future follow-up studies to facilitate longitudinal analysis (if funding is granted) on aging in a transforming societal context.

**International Registered Report Identifier (IRRID):**

DERR1-10.2196/41255

## Introduction

### Background

Inclusive and accessible living environments are fundamental to well-being, and Swedish aging policy states that older adults should be able to age safely, maintain their independence, and continue to lead active lives. However, with demographic change, globalization, and urbanization, health inequality is a growing societal challenge. The chances of older adults being able to lead a good life vary depending upon which of Sweden’s 290 self-governed municipalities they live in. There is a lack of knowledge on what it is like to age in areas where the societal challenges are most evident. International studies show that the quality of life of older adults living in disadvantaged areas is affected in many ways. They have worse physical and mental health, activity, and participation restrictions—and shorter life expectancy. In Sweden, research on how living in a disadvantaged area affects older adults’ quality of life is virtually nonexistent. We argue that disadvantaged areas exist in both urban and rural contexts, but conditions, experiences, and perceptions may be quite different. This study addresses how the home and neighborhood influence quality of life for older adults who live in urban and rural disadvantaged areas and how these person–context dynamics are experienced. In the proposed study, we will investigate urban disadvantaged areas with a high proportion of crime, social unrest, fast population turnover, and a high percentage of unemployment [1]. We will also investigate rural disadvantaged areas with a consistently decreasing population and a shrinking tax base for health and social care services [2]. We will study areas that are changing in many ways. Older adults tend not to move as often as younger adults, thus experiencing the consequences of disadvantaged areas in transition. By focusing on older adults, the results will aid in understanding how societies can ensure healthy lives, promote quality of life at all ages, and have sustainable cities and communities that provide opportunities for all.

### Disadvantaged Areas in Sweden

In 2016, the Swedish National Police published their first-ever report on what they called “socially disadvantaged areas”; the most recent report, from 2017, listed 61 areas in Sweden. According to the Swedish National Police, a socially disadvantaged area is a geographically defined urban area with low socioeconomic status, high levels of crime, fast population turnover, and a high proportion of immigrants. Residents are at risk of being excluded, feeling alienated, losing faith in the future, and having health problems. Over time, criminal networks have become increasingly present [1]. Typically, the neighborhoods are large residential areas with little traffic. Landlords are typically public housing companies, and the dwellings are rented apartments in multi-dwelling blocks. The areas have been criticized for not being well kept, needing maintenance and repairs, and being overcrowded. Lately, retail store owners have been reluctant to establish businesses in these neighborhoods, which reduces amenities. Approximately 81% of the population is of non–Swedish origin, and the proportion of older adults is smaller than the national average: approximately 12.5%, compared to 19% nationally. Of the Swedish-born population, the majority are likely older and have lived in the area for many years. For older adults, safety and security risks and not having access to local services are important concerns, as are feelings of alienation from the neighborhood. Contrastingly, other surveys show that many residents like their neighborhood and that crime rates are slowly decreasing [3]. Turning to rural areas, depopulated areas make up another supposedly disadvantaged area. Depopulated areas are municipalities or parts of municipalities that have been characterized by significant population decline for the last 20 years due to urbanization, high unemployment rates, and weak housing markets [2]. These areas also have a larger proportion of older adults than the national average: 25%, compared to 19% nationally, and due to a reduced tax base (ie, a high social dependency rate) public health and social care services are expensive to provide and facilities are sometimes closed. It is also difficult to provide small, suitable dwellings for older adults who want to downsize [2]. In these areas one finds that older adults mostly live in single-family houses, are homeowners, and were born in Sweden. Car dependency is high. Retail store owners have had a hard time keeping shops and businesses running, which reduces amenities in the area. It is likely that urban and rural disadvantaged areas are influencing older adults’ quality of life in ways that are unique to each, but also in other ways that are similar in both. An understanding of how the context influences older adults in both urban and rural disadvantaged areas is needed to reveal the heterogeneity of living conditions of older adults in Sweden.

Sweden could be described as a social democratic welfare state in transition. The Swedish population, and older adults in particular, are known to show high levels of trust toward government officials, media, and fellow citizens in general. The health care and social care systems have universal coverage, but local differences in service provision are a growing challenge to equality [4]. The wave of immigrants that entered Sweden in 2014 to 2015 has affected municipalities in rural areas (where many were placed upon arrival) and urban areas (where many settled by choice). With increasing societal challenges in Sweden and with new generations of non–Swedish born older adults living in disadvantaged areas, research on aging in Swedish disadvantaged areas can contribute to international knowledge on quality of life in later life in disadvantaged areas.

### Overview of the Research Field

In general, older adults tend to move less often than younger adults, and the home becomes an essential arena for social life with increasing age. Health and quality of life are likely more influenced by the home and neighborhood in older adults than in younger people [5]. Accessible homes support older adults in managing activities of daily living independently for longer [6]. High housing satisfaction is related to higher life satisfaction, and higher neighborhood social cohesion supports participation in activities and in society. However, we do not know whether previous findings from Swedish and European home-health-dynamics studies of older adults, which had participants who were fairly healthy and socioeconomically well-off (eg, the ENABLE AGE Project [7]), apply to older adults in disadvantaged areas. We do not know if previous findings on the importance of weak ties among neighbors apply within areas that are transitioning and have fast population turnover, as in disadvantaged areas in Sweden [8].

International reviews of older adults living in neighborhoods with low socioeconomic status (SES) show that a variety of neighborhood factors negatively impact depression, cognitive skills, and overall health [9-11]. Further, disadvantaged areas can restrict outdoor mobility possibilities for participation, and ultimately increase the risk of mortality [12-15].

Several authors conclude that there is a need for more nuanced measures of neighborhood quality, both objective and self-rated. It is also necessary to investigate contradictory results (eg, ethnic enclaves in the United States seem to have a positive effect on health for Latino populations but not for African American populations [11]) and to identify modifiable aspects of the living environment to develop interventions supporting quality of life. A meta-analysis of neighborhood effects on mortality included 11 studies from Sweden; however, none focused on older adults [15]. The authors found reduced life expectancy among residents from areas with low neighborhood SES, and they concluded that in order to better understand how social and physical neighborhood factors contribute to well-being, quantitative methods need to be complemented with qualitative methods.

Researchers on rural aging have explored the challenges older adults experience when the need for support increases, but community changes have resulted in health and social care services being closed. This research highlights the strong desire to age in place and the constant negotiations that come with such a decision.

Nevertheless, other researchers have questioned the social sustainability of depopulated areas, arguing that rural aging needs more attention [16] and that knowledge of how home and community factors interact with quality of life in rural areas is lacking.

It has been suggested that older adults are more dependent than younger adults on the home and neighborhood context and are more vulnerable to neighborhood change; however, this has not been empirically supported by recent studies. Contrastingly, it has also been suggested that older adults with lower SES might be more resilient to neighborhood stressors than older adults from areas with high SES [5]. Both of these conclusions are highly generalized, and it is thus important to explore positive aspects of living in disadvantaged areas and critically examine negative images from media to obtain a nuanced picture of the day-to-day life of older adults in disadvantaged areas.

### Theoretical Underpinnings

Acknowledging the complex individual–context dynamics and quality of life in disadvantaged areas calls for several theoretical perspectives. We ground our study in a theoretical framework of ecological theories on aging, social networks, human geography, occupational justice, and a perspective of the social life course [17-21].

Early models from Lawton and Nahemow [19] are based on an environmental gerontology perspective that usually includes aspects of the built environment and tries to explain the fit or the congruence between the capabilities of the person and the demands of the environment. We will use the concept of place-making [21], describing how older adults develop “insideness” and the process of belonging and identity in relation to a home or an area. After relocation to a new space, place-making skills might be disrupted if other changes occur as well. If the older adult cannot continue with their day-to-day habits and routines in the new dwelling, the older adult might not develop attachment.

We also add a social layer that includes identity, roles, and the norms that are created and formed within the social context. Moreover, being, acting, and meaning-making are interactive processes between the individual and the social context. The individual takes part in creating the context and the context creates the individual. The complexity of and the forces within social contexts need to be acknowledged to better understand the effects of exclusion and marginalization on disadvantaged areas and the effects of inclusion and creation of community in seemingly harsh areas [22].

The social life-course perspective explains that aging is contextual and that the journey of individuals across the life course is parallel to development and changes in the surrounding society [20]. Individuals age and change while the society they live in ages and changes, too. Older adults are likely to interpret feelings of well-being and belonging in their neighborhood in the light of ideas on the past, present, and future of their life course and the surrounding society. Further, social network theory and theories on social ties will be important in understanding how the context influences quality of life [8,18].

### Conceptual Definitions

#### Quality of Life

We use the World Health Organization Quality of Life Group definition of quality of life as a construct that captures an individual’s physical and psychological health, social relationships, and the environment and defines quality of life as an individual’s perception of their position in life in the context of the culture and value systems in which they live and in relation to their goals, expectations, standards, and concerns [23].

#### Participation

The World Health Organization defines participation as an individual’s involvement in life situations that let them take part in society [24], in addition to participation in tasks and activities that are meaningful to them (eg, occupations) and promote health, well-being, and participation in life [17].

#### Context

We define the environment surrounding the individual as their context, focusing on the home and neighborhood. This context includes physical factors (eg, buildings and spaces), social factors (eg, family, friends, neighbors, and social networks) and emotional or identity factors (eg, place attachment and belonging).

### Study Objective and Research Questions

The study objective is to investigate how home and neighborhood influence participation, quality of life, identity, and belonging of older adults living in urban and rural disadvantaged areas in Sweden, and how these person–context dynamics are experienced by older adults in transitioning neighborhoods. We have three research questions. First, how do older adults in disadvantaged areas reason about their home and neighborhood in relation to their quality of life, and how do they act on concerns regarding their neighborhoods? We will pay particular attention to ways that identity and belonging are manifested, ways that participants relate to the existing media images of the area they live in, participants’ thoughts on relocation, and how they place themselves in a transforming neighborhood context. Second, how are observable and self-rated home and neighborhood factors associated with the quality of life of older adults in disadvantaged areas? We will pay particular attention to urban/rural, gender, and Swedish born/non–Swedish born subgroup differences as potential mediators. Third, how can we contribute to existing theoretical perspectives on aging in context by contrasting and integrating knowledge of older adults’ quality of life in urban and rural disadvantaged areas?

## Methods

### Study Design

This baseline study has an explanatory sequential mixed methods design [25] and includes 3 phases. Phase 1, the interview study, includes semistructured interviews and photo-elicitation interviews with 40 subjects). Phase 2, the survey study, will collect quantitative data via telephone survey interviews; 400 subjects will be recruited and added to the 40 phase-1 subjects, for a total of 440 subjects. In phase 3, the integration study, data from the previous phases will be combined and analyzed with a mixed methods analysis approach [25].

### Population and Setting

The study population is community-living adults aged 65 years and older who have lived in any of the targeted disadvantaged areas for at least 5 years.

#### Urban Areas

The urban areas targeted (N=5) are located in 2 smaller cities in the south of Sweden (with populations of 46,000 and 150,000) and are seen as typical examples of how multi-dwelling neighborhoods that were built in the 1960s and 1970s have transitioned over time into disadvantaged neighborhoods, as defined by the Swedish National Police. In recent years, efforts have been made to increase police presence to reduce crime rates and improve safety. In one area, city officials have taken drastic measures to improve housing standards by demolition and new construction, and the neighborhood transition is characterized by gentrification—so far, a rare approach in Sweden.

#### Rural Areas

The rural areas targeted are rural municipalities in the south of Sweden (with 10,000 to 15,000 inhabitants). The municipalities have been characterized by depopulation for approximately the last 20 years, with the exception of 2015 to 2016, when a large influx of immigrants changed the population structure and increased the population. However, the dependency ratios are still unfavorable. The dependency ratios in these 2 municipalities are between 87% and 91%, compared to 77% in both the south region, Skåne, and in Sweden in general. The municipalities are characterized by several smaller villages, among which there is a “municipality capital” that is usually of similar size to the other villages. Unlike rural municipalities in the central and northern parts of Sweden, these municipalities are within 45 minutes by car of a larger city.

### Phase 1: Interview Study

#### Recruitment

Participants were recruited via community centers serving older adults, libraries, and nonprofit organizations located in the targeted areas. Because of the COVID-19 pandemic, we had to adapt our strategy. Thus, recruitment was also done by mail, after retrieving the addresses of all adults over the age of 65 in each disadvantaged area from the Swedish state personal address register (SPAR). A set of 20 randomly chosen residents received a letter with information about the study that was followed up with a telephone call, a procedure that will be repeated if needed. We aimed for a sample of N=40.

#### Data Collection

The data collection included semistructured interviews for which we developed an interview guide (Multimedia Appendix 1) based on the aim of the study. At the end of the interview, the participants were instructed to take photographs of the area they lived in and defined as their neighborhood. In urban areas, this was usually the block or the street they lived on, while in the rural areas, the participants usually referred to their village or the neighboring houses. They were instructed to take photographs of places, buildings, or things that they considered important to themselves, either positively or negatively. At the second interview, according to the photo-elicitation technique, the interview focused on the content of the pictures. The interview guide and the data collection procedure were pilot-tested with 3 participants. Only minor adjustments to the interview guide were needed, and the 3 pilot-test participants were thus included in the final sample. Due to the COVID-19 pandemic, interviews were conducted either face-to-face at home, at a community center serving older adults, outside in the garden, in the neighborhood, or remotely via video conferencing software or telephone.

#### Data Analysis

Data will be analyzed with different qualitative data-analysis approaches, such as the thematic analysis described by Braun and Clarke [26]. Both inductive and deductive approaches will be used, depending on the research question.

### Phase 2: Survey Study

#### Power Calculation, Sample Size and Recruitment

We aim for a sample size of N=400 in phase 2. Power calculations were made based on the World Health Organization 10 Wellbeing index [27], assuming a mean difference of 4.0 with an SD of 10.0 (power 0.80 and *P*<.05), resulting in a recommended sample of at least 98 participants per area. Acknowledging that the power calculation was highly indicative, that the study design was explorative overall, and that we aimed to perform follow-up data collection (if granted funding), we decided to include 200 participants from urban areas and 200 participants from rural areas. To recruit them, we ordered lists of contact information for all adults aged 65 years or older in the targeted urban and rural areas from the SPAR and performed randomization. We will aim for a sample that includes 50% urban participants and 50% rural participants, with 50% being aged 65 to 79 and 50% aged 80 or older. In all, approximately 12,500 adults 65 years or older live in the targeted areas. Anticipating an inclusion rate of 20% or more, the randomized lists will contain a total of 2000 names. Our procedure is to send out information letters and follow up with a telephone call. If the participant meets the inclusion criteria and is willing to participate, we set a date for the telephone interview.

#### Survey and Data

The survey was developed based on the aim of the study and contains standardized instruments and study-specific questions on quality of life, participation, and health, as well as physical, social, and emotional aspects of housing and neighborhood. Sociodemographic information and information on possible confounders will also be collected. Table 1 shows an overview of all standardized instruments and study-specific questions used in the survey. Study-specific questions and instruments that we adapted for the study are described below [28-35].

Housing satisfaction is measured with 7 study-specific questions regarding housing standards, including size, design, internet, parking, storage, and accommodation of guests, as well as an overall question: “In general, how satisfied are you with your current housing situation?” The questions are answered on a scale from 0 (extremely dissatisfied) to 10 (extremely satisfied). Five questions concern the extent to which the bathroom, kitchen, entrance, bedroom, and living room are practical to use. The questions are answered on a scale from 0 (extremely impractical) to 10 (extremely practical). Five questions concern different aspects of safety at home. The questions are answered on a scale from 0 (extremely unsafe) to 10 (extremely safe). The questions were inspired by a subset of questions from an earlier version of the online self-help tool “Housing Options for Older People” [36,37]. The respondents also respond to 3 statements on housing discomfort with the responses “very true,” “partly true,” or “not true at all.” The statements are as follows: “I often feel alone in my home,” “I often feel that I cannot be left alone/in peace in my home,” and “I often feel bored in my home.”

To capture the emotional aspects of the neighborhood, we use the Person-Place Fit Measure for Older Adults (PPFM-OA), developed by Weil [28]. We used a dual-panel approach to translate the measure into Swedish [38]. In brief, panel 1 consisted of 2 registered occupational therapists with PhD degrees and experience working in both Swedish and US contexts with similar target groups as this study. They individually translated the items, discussed them, and then agreed on a translation. Disagreements, as well as translations that were problematic due to cultural and contextual differences between the United States and Sweden, were discussed with the developer, as well as with an academic panel consisting of 9 graduate students and junior housing and aging researchers. Panel 2 consisted of a selection of potential end responders: 4 older adults (age range 74 to 88 years) who provided feedback on the items and the structure of the translated tool using cognitive interview techniques. After panel 2, final revisions were made and 3 items were excluded due to cultural differences regarding health care and housing options. The participants respond to 41 statements using a Likert scale ranging from 5 (strongly agree) to 1 (strongly disagree).

For the presence and use of services in the neighborhood, we use the Participation in Activities and Places Outside Home Questionnaire (ACT-OUT). ACT-OUT was developed to explore social citizenship through out-of-home participation in activities and places for older adults with and without dementia. We use the first part of ACT-OUT, which registers whether or not the respondent uses 24 types of services and places, including consumer, administrative, and self-care places, places for medical care, social, cultural, and spiritual places, and places for recreational and physical activities [29]. For the current study, we added an initial question on presence—“Does the service exist in your neighborhood?”—before questions regarding previous and current use and the desire for future use of the services listed in ACT-OUT.

For social interaction and activities, the participants respond to questions on how often they do any of 13 activities. The activities include different forms of interacting with friends, relatives, and neighbors, participating in leisure activities, exercising with others, engaging with nonprofit organizations, participating in religious events, discussing or engaging in local politics, and participating in adult education and study groups. Responses include the following: “every day,” “1 to 2 times a week,” “1 to 2 times a month,” “1 to 2 times a year,” and “less than once a year or never.”

**Table 1:** Survey overview of instruments and study-specific questions.

Area/focus			Instrument		Source
**Quality of life**					
	Quality of Life	World Health Organization Quality of Life Assessment—Brief Scale		World Health Organization Quality of Life Group 1998 [23]	
**Dwelling**					
	Year of build	Study-specific		N/A^a^	
	Number of rooms	Study-specific		N/A	
	Number of bathrooms	Study-specific		N/A	
	Number of levels	Study-specific		N/A	
	Garden/balcony	Study-specific		N/A	
	Housing satisfaction	Study-specific^b^		N/A	
	Housing safety	Study-specific^b^		N/A	
	Housing discomfort	Study-specific		N/A	
**Neighborhood**					
	Neighborhood satisfaction/age-friendliness	Person-Place Fit Measure for Older Adults		Weil 2020 [28]	
	Presence and use of services	Participation in Activities and Places Outside Home for Older Adults^c^		Margot-Cattin 2019 [29]	
**Participation**					
	Participation	World Health Organization Disability Assessment Schedule 2.0 (section 6)		Üstün et al 2010 [30]	
	Social networks	Survey of Health, Ageing and Retirement in Europe		Litwin et al 2013 [31]	
	Social interactions and activities	Study-specific		N/A	
	Caregiving	Survey of Health, Ageing and Retirement in Europe^d^		Börsch-Supan et al 2005 [32]	
**Health and disease**					
	Disease	Survey of Health, Ageing and Retirement in Europe		Börsch-Supan 2019 [33]	
	Self-rated health	SF-36		Sullivan et al 1994 [34]	
	Pain	Study-specific		N/A	
	Falls	Study-specific		N/A	
	Hospital stays	Study-specific		N/A	
	Activities of daily living	ADL^e^ staircase		Iwarsson et al 2009 [35]	
	Receiving care	Survey of Health, Ageing and Retirement in Europe^d^		Börsch-Supan et al 2005 [32]	
**Sociodemographics**					
	Age	Study-specific		N/A	
	Sex	Study-specific		N/A	
	Number of years living in the neighborhood	Study-specific		N/A	
	Cohabiting	Study-specific		N/A	
	Marital status	Study-specific		N/A	
	Country of origin	Study-specific		N/A	
	Education	Study-specific		N/A	
	Work	Study-specific		N/A	
	Income	Study-specific		N/A	
	Housing supplement	Study-specific		N/A	
	Type of dwelling	Study-specific		N/A	
	Housing tenure	Study-specific		N/A	
	Access to car	Study-specific		N/A	

^a^N/A: not applicable.

^b^These questions were inspired by a subset of questions from an earlier version of the online self-help tool “Housing Options for Older People” [36,37].

^c^We added a question on presence—“Does the service exist in your neighborhood?”—prior to questions regarding use of services included in the Participation in Activities and Places Outside Home for Older Adults instrument [29].

^d^These questions were based on the Survey of Health, Ageing and Retirement in Europe [32], but were modified.

^e^ADL: activities of daily living.

For receiving and giving care we use a modified set of questions from the Survey of Health, Ageing and Retirement in Europe (SHARE) [32]. The respondents are asked whether they have received personal care or help with household chores, household maintenance, paperwork, translations, computers and the internet, or transportation in the last 12 months as (1) informal care from someone in the household; (2) informal care from someone outside the household; or (3) formal care. They are also asked whether they have a companion at medical visits. Then, the respondents are asked if the formal or informal care from within or outside the household was received approximately daily, weekly, monthly, or less often. For caregiving, we asked whether the individual had, in the last 12 months, given informal care within or outside the household in the 7 areas mentioned above. The respondents are also asked to estimate how often they provided care.

The survey also asked the participants about pain, as follows: “Have you in the last 30 days been bothered by pain? If yes, how bad is the pain most of the time (mild, moderate, or severe)?” We then asked a question about where in their body they experienced pain. The survey also includes questions about whether or not the respondent has fallen in the last 12 months, where falls occurred, and if the respondent needed medical care due to falls. We also ask about the number of overnight hospital admissions in the last 12 months, regardless of cause.

#### Data Collection

Data collection was intended to take place at home. However, due to COVID-19 restrictions at the time, we changed this to telephone interviews. The participants return the signed consent form by mail before the phone interview takes place. Questions and scales are mailed to the participants to serve as visual aids during the interview. Answers are recorded by the data collector using RedCap software (Research Electronic Data Capture; Vanderbilt University). Interviews can be completed in Swedish, English, Arabic, Persian, Slavic languages, Polish, or Danish if needed. We will use an interpreter when needed.

#### Data Quality

Besides the research team doing the interviews (who all have a bachelor’s degree or PhD in health science or social science), the data collectors are students in an occupational therapy bachelor’s program. All data collectors have received study-specific training. At the start of the study, 3 data collectors performed 2 interviews each (n=6), after which data collection was paused and the data collectors and research teams engaged in a thorough discussion and evaluation. Minor alterations to and clarifications of the instructions were edited into RedCap before the data collection continued. Frequent meetings with the data collectors and the principal investigator of the study will be held throughout the data collection period.

### Statistical Data Analysis

For newly translated measures not before used in this context (eg, PPFM-OA [28]), preparatory analyses targeting validity and precision will be conducted before proceeding to primary analyses targeting specific research questions. The survey data will be analyzed with mainstream statistical analyses and SEM to explore and evaluate interactions between quality of life, home and neighborhood factors, and sociodemographic factors. The use of SEM will also support and question theory building.

### Phase 3: Integration

In accordance with a mixed methods approach [25,39], phase 3 will be an analytical step comprising integration and synthesis of the data and results from previous phases. This synthesis will generate new knowledge by comparing, contrasting, and positioning a diversity of findings with each other. Data presentation workshops and analysis meetings with researchers, participants, and knowledge users will be the core methodology. Emerging themes will be contrasted against theoretical and empirical scholarly work on aging in context in an iterative process. Preliminary findings will be discussed in a series of workshops with scholars in the field, participants from our study, and potential knowledge users. The integration task is expected to be completed after 3 to 6 months.

### Ethics Approval

All participants will sign written consent forms when entering the study. The study was approved by the Swedish Ethical Review Authority (phase 1: Dnr. 2020-03468; phases 2 and 3: Dnr. 2021-03588).

## Results

As of the submission of this protocol (August 2022), recruitment for the interview study has been completed (N=39), and 267 participants have been recruited and completed data collection for the survey study. We expect recruitment and data collection to be finalized by December 2022.

## Discussion

### Anticipated Findings

This study will provide detailed knowledge on what role the home and neighborhood play in older adults’ quality of life and their participation in the community and society by focusing on different kinds of disadvantaged areas. By collecting qualitative and quantitative data and addressing physical, social, and emotional aspects of the environment, we will be able to address complex person–environment dynamics [39].

In Sweden, the development of and changes in disadvantaged areas in cities and depopulated areas in rural municipalities are caused by shared challenges on a societal level, such as demographic changes, urbanization, and globalization, but also due to local challenges in Sweden’s 290 self-governing municipalities [4]. By including urban and rural areas in this study, we will be able to investigate both similarities and differences in the person–environment dynamics of older adults’ quality of life and participation in the community and society.

The study design allows for studying older adults not only as they are affected by the environment, when the neighborhood changes and they have to adapt, but also as active agents, who likely contribute to change in the neighborhood and build the community. The knowledge generated will be useful to better understand the effects of exclusion and marginalization and the effects of inclusion and the creation of community [22].

Inclusive and accessible living environments are fundamental to well-being, and Swedish aging policy states that older adults should be able to age safely, maintain their independence, and continue to lead active lives. The right to be active, live independently, and participate in the community and society can be considered an occupational justice issue, not only an individual health matter, and is important for future research and policy interventions on healthy aging [17].

### Limitations

The disadvantaged areas selected for the study are not representative of all disadvantaged areas in Sweden, but they constitute interesting examples that show the diversity of living conditions of older adults. The study was started in 2019, and recruitment, as well as quantitative and qualitative data collection, had to adjust to the COVID-19 pandemic. This likely also influenced the data collected, which we will consider in future analyses and interpretation of results.

### Conclusions

With an increasing proportion of older adults, an increasing number of disadvantaged areas, and an increasing dependency ratio in more than 50% of Swedish municipalities, Swedish cities and municipalities are transforming and becoming increasingly segregated. This study will add unique knowledge on what it is like to be older in disadvantaged areas and deepen knowledge on housing and health dynamics in later life. Further, the design of the current study will allow future follow-ups and longitudinal analysis (if granted funding) of aging in a transforming societal context.

## Appendix

Multimedia Appendix 1 Interview Guide.

Multimedia Appendix 2 Peer-review reports from the Forskningsrådet för hälsa, arbetsliv och välfärd (FORTE, Swedish Research Council for Health, Working Life, and Welfare) (Stockholm, Sweden).

## Abbreviations

ACT-OUT: Participation in Activities and Places Outside Home Questionnaire
PPFM-OA: Person-Place Fit Measure for Older Adults
SEM: structural equation modeling
SES: socioeconomic status
SHARE: Survey of Health, Ageing and Retirement in Europe
SPAR: Swedish state personal address register

## Data Availability

The data sets generated during the current study will be made available after data collection is completed upon reasonable request to the corresponding author.

## References

[1] (2017). Deprived areas: Social order, criminal structures and challenges for the police force. Utsatta områden - Social ordning, kriminell struktur och utmaningar för polisen. Swedish National Police.

[2] Housing for the elderly in depopulated areas. Swedish National Audit Office.

[3] Ivert A-K, Torstensson Levander M, Mellgren C (2016). The unequal safety net. Den ojämlika tryggheten. Socialvetenskaplig tidskrift.

[4] Jönson H, Szebehely M (2018). Eldercare in Sweden: Local variation and general trends. Äldreomsorger i Sverige: Lokala variationer och generella trender.

[5] Schafer (2018). (Where) is functional decline isolating? disordered environments and the onset of disability. J Health Soc Behav.

[6] Iwarsson S, Horstmann V, Slaug B (2007). Housing matters in very old age - yet differently due to ADL dependence level differences. Scand J Occup Ther.

[7] Iwarsson S, Löfqvist C, Oswald F, Slaug B, Schmidt S, Wahl H, Tomsone S, Himmelsbach I, Haak M (2016). Synthesizing ENABLE-AGE research findings to suggest evidence-based home and health interventions. J Hous Elderly.

[8] Henning C, Lieberg M (1996). Strong ties or weak ties? Neighbourhood networks in a new perspective. Scand Hous Plan Res.

[9] Julien D, Richard L, Gauvin L, Kestens Y (2012). Neighborhood characteristics and depressive mood among older adults: an integrative review. Int Psychogeriatr.

[10] Wu Y, Prina AM, Brayne C (2015). The association between community environment and cognitive function: a systematic review. Soc Psychiatry Psychiatr Epidemiol.

[11] Yen IH, Michael YL, Perdue L (2009). Neighborhood environment in studies of health of older adults: a systematic review. Am J Prev Med.

[12] Rosso A, Auchincloss A, Michael Y (2011). The urban built environment and mobility in older adults: a comprehensive review. J Aging Res.

[13] Danielewicz AL, Dos Anjos Juliana Cristine, Bastos JL, Boing AC, Boing AF (2017). Association between socioeconomic and physical/built neighborhoods and disability: A systematic review. Prev Med.

[14] Annear M, Keeling S, Wilkinson T, Cushman G, Gidlow B, Hopkins H (2012). Environmental influences on healthy and active ageing: a systematic review. Ageing Soc.

[15] Meijer M, Röhl Jeannette, Bloomfield K, Grittner U (2012). Do neighborhoods affect individual mortality? A systematic review and meta-analysis of multilevel studies. Soc Sci Med.

[16] Abramsson M, Hagberg J (2018). What about community sustainability? – dilemmas of ageing in shrinking semi-rural areas in Sweden. Scottish Geogr J.

[17] Durocher E, Gibson B, Rappolt S (2013). Occupational justice: a conceptual review. J Occup Sci.

[18] Granovetter MS (1973). The strength of weak ties. Am J Sociol.

[19] Lawton P, Nahemow L, Eisdorfer C, Lawton P (1973). Ecology and the aging process. The psychology of adult development and aging.

[20] Riley M (1987). On the significance of age in sociology. Am Sociol Rev.

[21] Rowles G, Watkins J, Warner Schaie K, Wahl H-W, Mollenkopf H, Oswald F (2003). History, habit, heart and hearth: on making spaces into places. Aging independently: Living arrangements for mobility.

[22] Phillipson C (2018). The Role of Community and the Environment in the Lives of Older People. Annu Rev Gerontol Geriatr.

[23] WHOQOL Group (1998). Development of the World Health Organization WHOQOL-BREF Quality of Life Assessment. Psychol Med.

[24] (2001). International Classification of Functioning, Disability and Health. World Health Organization.

[25] Creswell JW, Clark VLP (2017). Designing and conducting mixed methods research.

[26] Braun V, Clarke V (2006). Using thematic analysis in psychology. Qual Res Psychol.

[27] Tinghög Petter, Carstensen J (2010). Cross-cultural equivalence of HSCL-25 and WHO (ten) Wellbeing index: findings from a population-based survey of immigrants and non-immigrants in Sweden. Community Ment Health J.

[28] Weil J (2020). Developing the person-place fit measure for older adults: broadening place domains. Gerontologist.

[29] Margot-Cattin I, Kuhne Nicolas, Kottorp Anders, Cutchin Malcolm, Öhman Annika, Nygård Louise (2019). Development of a questionnaire to evaluate out-of-home participation for people with dementia. Am J Occup Ther.

[30] Ustün T Bedirhan, Chatterji S, Kostanjsek N, Rehm J, Kennedy C, Epping-Jordan J, Saxena S, von Korff M, Pull C, WHO/NIH Joint Project (2010). Developing the World Health Organization Disability Assessment Schedule 2.0. Bull World Health Organ.

[31] Litwin H, Stoeckel K, Roll A, Shiovitz-Ezra S, Kotte M, Malter F, Börsch-Supan A (2013). Social network measurement in SHARE wave four. SHARE Wave 4, innovations and methodology.

[32] Börsch-Supan A, Jürges H (2005). The Survey of Health, Aging, and Retirement in Europe – Methodology. SHARE Project.

[33] Börsch-Supan A Survey of Health, Ageing and Retirement in Europe (SHARE) Wave 6. SHARE Project.

[34] Sullivan M, Karlsson J, Ware JE (1994). SF-36 hälsoenkät : svensk manual och tolkningsguide (Swedish manual and interpretation guide).

[35] Iwarsson S, Horstmann V, Sonn U (2009). Assessment of dependence in daily activities combined with a self-rating of difficulty. J Rehabil Med.

[36] Heywood F, Oldman C, Means R (2002). Housing and Home in Later Life.

[37] FirstStop Advice. Housing Options (HOOP) Tool.

[38] Hagell P, Hedin P, Meads DM, Nyberg L, McKenna SP (2010). Effects of method of translation of patient-reported health outcome questionnaires: a randomized study of the translation of the Rheumatoid Arthritis Quality of Life (RAQoL) Instrument for Sweden. Value Health.

[39] Weil J (2017). Research design in aging and social gerontology: Quantitative, qualitative, and mixed methods.

