# Supplementary material for: Older Adults Living in Disadvantaged Areas: Protocol for a Mixed Methods Baseline Study on Homes, Quality of Life, and Participation in Transitioning Neighborhoods
Source: JMIR Res Protoc. 2022 Oct 12;11(10):e41255. doi: 10.2196/41255 (PMC9607888; doi:10.2196/41255)
Supplement: Multimedia Appendix 1 [file resprot_v11i10e41255_app1.pdf]

SUPPLEMENT: INTERVIEW GUIDE

Interview Questions

1. Please, let us start with you telling me about yourself and what it is like to live in this neighborhood?

2. When I say "neighborhood", how do you define it? What is your neighborhood? Is it [name of the deprived area]? Or is your neighborhood only a part of that? Who do you consider your neighbors?

3. Why do you live here? Would you like to share with me, the story behind your decision to move here?

4. The police labeled your neighborhood a deprived neighborhood. It is in the news from time to time too. Did you know of it? Have you seen such news? What do you think of it? Do you agree? Is it fair? What do you think when you read about your local area in the newspaper?

5. You said you had lived here for [xx] years. Have you changed since you moved here? (Health, growing older, changed roles, changed habits?)

6. What do you think about your health status? Do you think your neighborhood influences your health or ability to do things and to socially participate?

7. Let´s turn to your apartment. How do you feel about your apartment? Do you feel at home here - or where would you feel at home? Is it practical/functional for you?

8. How do you think people who do not live, think and talk about your neighborhood? What do you think about it?

9. How would you describe the atmosphere in the neighborhood? Do you consider your neighborhood being a community? How so? Are you a part of it? In what ways? How are neighbors treating each other? Do you think of your neighbors as your friends?

10. What does this place mean to you? Throughout life, have there been other places that were meaningful to you? Can you rate them in relation to each other? Has it changed (over time)?

11. Do you think that your neighborhood has changed since you moved here and if so, how? Are there things that have not changed?

12. Have the changes you mentioned influenced your daily activities outside the home in any way? Your level of independence? Social participation?

13. Are there activities that you wish you could do, or places you want to visit in the neighborhood that you cannot due to the situation in the area? What would you miss the most if you moved away?

14. Is there something you would like to change in this area?

15. Are you involved in any community groups, civic engagement? How do you think each resident living here can contribute a sense of community?

16. How do you see the years ahead? Will you stay here? Move? Where do you see yourself in 5 years?

17. What do you think about the future of the neighborhood? Where do you see the neighborhood in 5 years?
