# Supplementary material for: Older Adults Living in Disadvantaged Areas: Protocol for a Mixed Methods Baseline Study on Homes, Quality of Life, and Participation in Transitioning Neighborhoods
Source: JMIR Res Protoc. 2022 Oct 12;11(10):e41255. doi: 10.2196/41255 (PMC9607888; doi:10.2196/41255)
Supplement: Multimedia Appendix 2 [file resprot_v11i10e41255_app2.pdf]

FORTE: Forskningsrådet för hälsa, arbetsliv och välfärd

Statement from the panel

PLEASE NOTE: This is a translation. Original PDF document was exported to Word and translated with Google translate by M Granbom July 20, 2022

**2019-00107**	**Marianne Granbom**	**Panel numer:**	**SOA19**

Funding initiative: Research project 2019

Funding form: Project Junior researchers

Project title (Swedish): Being older and living in vulnerable areas. A study with mixed methods about the importance of the home and the surrounding area for well-being in changing urban and rural environments.

Focus: Free

Evaluation

Purpose, issues, theory connection, background and the project's originality

This project aims to better understand the situation of the elderly in different types of vulnerable areas, in urban environments and rural areas. How the housing situation in these areas relates to health and well-being will be studied using different methods. A questionnaire study in three residential areas is followed up with qualitative interviews that will use photos. These two parts are finally synthesized or integrated into the theoretical contribution of this study. Although vulnerable areas have been studied, it is unusual to focus on the situation of the elderly and it is unusual to compare such different types of vulnerable areas.

The questions are relevant and the need to know more about the subject is great. The theoretical background deals with, among other things, how surroundings can be seen from different perspectives and that the individual's interaction with his surroundings is what creates meaning. The environment is expected to affect well-being differently depending on where one lives, gender and background. Family, social networks are included here in the environment but are not specifically discussed as more decisive than other factors. Three areas in Skåne are studied here. The project does not claim to be representative of other areas in Sweden, where conditions may look completely different, but the specifics of these selected areas in Skåne are not discussed. How the situation in urban and rural areas with vulnerability can be compared, and why this is important, could be made clearer.

Study design, methods for data collection and analysis

The study will be conducted with a questionnaire and home visits, with an interpreter if needed. Due to anticipated difficulties in getting respondents, a three times as large sample will be drawn. This is an ambitious approach, but a discussion about whether selection in the respondent is foreseen would be in order. The study hopes to lead to 360 questionnaire responses. Home visits are costly but probably necessary for this group of respondents. Those who are excluded due to various factors (such as language) will probably be solved in the specific situations and there are advantages to visitor interviews as the interviewer has greater control over what and how answers are collected.

The subsequent in-depth interviews are not described in detail and the reader gets the impression that its form will settle down after the questionnaires have been collected. Of course, this involves a risk factor, but also the opportunity to ensure that the two parts fit well together. The three residential areas include an area with great change and how this is characterized and what function this area fulfills could be specified better. The motives for choosing areas can be expressed more clearly. However, the method seems reasonable and well described, even if the third phase is difficult to grasp.

Gender and diversity perspectives in the content of the research

Gender and diversity are central to the project and are well discussed. It is likely that language difficulties may occur in some areas where many older people born abroad live and this is reflected on. The approach with two such different types of areas is innovative and it is discussed how these areas can have different effects on well-being for different groups. Given the diversity that probably exists among the respondents, it is not likely that anything validated can be said about different groups, but the situation will begin to be described in its variation, if the project is successful.

Feasibility

What can be seen as a risk in the study is to find respondents who are willing to participate in the interview. Especially to get the spread in experience and background that the researchers intend. The qualitative interviews will be based on photographs taken by the respondents from their surroundings and a discussion about this. Of course, both technical and other problems can arise, that the discussion does not lead to the type of information the researchers are looking for. The method is innovative and exciting, but places great responsibility on the respondents' active participation and compliance. Those who choose to say yes to this may be active people with a great interest in their surroundings.
The interviews will be conducted by two of the applicants, a doctoral student and a research assistant. The fact that several people and also the experienced researchers are involved in the interviews can ensure that solutions are found for situations that arise during data collection.

Relevance, collaboration and utilization

The study has great societal relevance and what the results can be used for is discussed. Knowledge of the elderly's relationship to their surroundings is needed in a Sweden with a crisis housing market. Data collection will also be performed so that follow-ups and extended studies can be done. Collaboration and utilization are described well and thoughtfully so that the results will benefit more people and this continuously.

Summary of the evaluation

In summary, the study is innovative and very interesting and deals with an important area where a lack of information and knowledge is great. It also has an interdisciplinary approach that is likely to advance knowledge. The project can be seen as exploratory and containing certain risk elements, but also elaborated and well planned. The study is not representative but can lead to both further studies and comparisons of vulnerable areas. Method development should also be possible.

Proposed decision (bevilja, bevilja i mån av medel, avslå/approve, approve subject to funding, reject)

Approve subject to funding.
